# Impact of intrapartum antibiotics on the developing microbiota: a review

**DOI:** 10.20517/mrr.2022.04

**Published:** 2022-07-21

**Authors:** Silvia Arboleya, Silvia Saturio, Miguel Gueimonde

**Affiliations:** ^1^Department of Microbiology and Biochemistry of Dairy Products, IPLA-CSIC, Villaviciosa 33300, Spain.; ^2^Diet, Human Microbiota and Health Group, Institute of Health Research of the Principality of Asturias (ISPA), Oviedo 33011, Spain.

**Keywords:** Neonate, microbiota, intrapartum, antibiotics, infant

## Abstract

The perinatal period sets the basis for the later physiological and immune homeostasis of the individual, with the intestinal microbiota being an important contributor to driving this homeostasis development. Therefore, the initial establishment and later development of the microbiota during early life may play a key role in later health. This early establishment of the intestinal microbiota is known to be affected by several factors, with gestational age, delivery mode, and feeding habits being extensively studied ones. Other factors are not so well understood, although knowledge has been accumulating in the last years. Among them, a factor of great relevance is the effect of perinatal exposure to antibiotics. Administration of intrapartum antimicrobial prophylaxis (IAP) to women during the delivery process represents the most common form of exposure to antibiotics during the perinatal period, present in around 30% of deliveries. During the last decade, evidence has accumulated demonstrating that IAP alters intestinal microbiota development in neonates. Moreover, recent evidence indicates that this practice may also be altering the infant intestinal resistome by increasing the levels of some antibiotic resistance genes. This evidence, as reviewed in this manuscript, suggests the interest in promoting the rational use of IAP. This practice has significantly reduced the risk of neonatal infections, but now the accumulating knowledge suggests the need for strategies to minimize its impact on the neonatal microbiota establishment.

## INTRODUCTION

The microbial colonization of the infant gut during the perinatal period is critical for the later health of the baby since the interplay of the microbiota with the host is a major force for the proper development of the homeostatic systems^[1]^. Thus, early colonization is key for later health, constituting a “window of opportunity” for microbiota modulation towards a healthy composition with long-term beneficial effects, as suggested by the “microbiota hypothesis”^[2]^.

Initially, the process starts with the settlement of facultative anaerobes, such as enterobacteria, and aerotolerant microorganisms. These microbes reduce the oxygen level in the gut environment allowing the multiplication of strict anaerobes, such as *Bifidobacterium* or *Bacteroides*, later followed by the increase of different clostridia members until, at the age of three years, the microbiota reaches a composition close to that found in adults, although not totally the same^[3]^.

This initial colonization of the infant is highly dependent on different factors, with gestational age at delivery, mode of delivery, and infant feeding habits being the most extensively studied ones^[4,5]^. Another factor also known to influence this early colonization process is exposure to perinatal antibiotics. The most common form of exposure to antibiotics at the perinatal stage is intrapartum antimicrobial prophylaxis (IAP)^[6,7]^.

Some decades ago, group B streptococci (GBS) was the neonatal pathogen showing higher morbidity and mortality, with mortality rates reaching 50% of infected babies. Then, in the 1980s, it was evidenced that administration of antibiotics to the mother during delivery was efficacious for preventing neonatal GBS infection. Therefore, recommendations on the use of IAP to reduce the risk for maternal-filial transfer of GBS were issued since this pathogen was common, being present in approximately 10%-30% of women^[8]^. This has been a successful approach, with the cases of GBS infection dropping about ten times since then^[8,9]^. The decision on whether and how to administer IAP to mothers is managed in different ways in different countries; some perform a prenatal screening for GBS carriage and only treat the positive mothers with IAP, whereas other countries use risk factor (prolonged rupture of membranes, intrapartum fever, *etc.*) assessment to decide on whether to administer IAP. Most often, IAP consists of the intravenous administration of penicillin or, alternatively, ampicillin. During the last years, the identification of the potential ability of IAP to induce alterations in the gut colonization process, together with interest in reducing antibiotic use, have attracted attention to the study of the impact of this prophylaxis. This is especially relevant since IAP is present in more than 30% of total vaginal deliveries^[6]^. Moreover, IAP is also common due to C-section practices, being recommended in all C-section deliveries since it reduces by more than half the complications of this surgery^[10]^. In this case, the antibiotic most commonly administered is cefazolin, except in the case of allergy^[11]^. 

The present review summarizes the current works in the gut microbiota field, analyzing IAP as an impact factor on its composition and function as well as the antibiotic resistance gene (ARG) load. A comprehensive search was conducted in the PubMed database. The search strategy combined the terms “gut microbiota”, “intestinal microbiota”, “neonate”, “infant”, “intrapartum antimicrobial prophylaxis”, “IAP”, “intrapartum antibiotics”, “delivery”, “group B streptococci”, “GBS”, “resistome”, and “antibiotic resistance genes”. References from included studies matching the inclusion terms, but not found with the search strategy used, were also included. Searches were limited to studies on humans, written in English, and published papers. No offspring age limit or date of publication restriction was used. The following data were extracted: year of study publication, country, characteristics of study population such as sample size (IAP group and control group, if it existed), delivery mode, gestational age at birth, antibiotic administration at delivery, time-point of stool collection, microbiota analyses methodology, gut microbiota composition, and resistome results.

## IMPACT OF IAP ON THE DEVELOPING MICROBIOTA COMPOSITION

Antibiotics are one of the main factors affecting the correct gut microbiota development and stability^[12]^. Different studies have focused on the effect of antibiotics directly administered to the individual; however, until recently, data on how antibiotics administered to mothers during delivery could affect the early microbiome colonization of the infant were scarce. In 2004, the first paper comparing the colonization patterns of gut microbiota in neonates born to mothers with and without amoxicillin for GBS prophylaxis was published^[13]^. This study found that only *Clostridium*, of the six bacterial groups analyzed by stool culturing, showed statistical differences among the groups, with lower counts in the IAP group of babies. No differences in other groups, such as *Bifidobacterium*, *Bacteroides*, or enterobacteria, were observed in this culture-based study. The topic was then forgotten until 2014, when Aloisio *et al.* published a paper using qPCR, a culture-independent technique^[14]^. They observed a negative impact of ampicillin for GBS prophylaxis in genus *Bifidobacterium* in seven-day-old infants’ feces, but the other bacterial groups analyzed were not significantly affected by the maternal treatment. It was not until 2015 that when a study using massive sequencing techniques of the 16S rRNA gene became available, reporting the effect of IAP upon the microbiota in a study with neonates born prematurely^[15]^. The authors found that the impact of IAP on the developing gut microbiota was even higher than the effect of direct administration of antibiotics to babies during the first days of life. Since then, more than 20 papers specifically examining the relationship between the IAP and gut microbiome have been published.

In the observational cohort of very low birth weight babies study conducted by Arboleya *et al.*, the direct administration of antibiotics to infants during the first days of life and/or to mothers during labor were compared^[15]^. The results show that, at one month of age, babies from IAP mothers and without other antibiotic exposure harbored lower relative abundances of different bacterial families, such as *Bifidobacteriaceae*, *Streptococacceae*, and *Staphylococcaceae*, and higher percentages of *Enterobacteriaceae*; these data were further confirmed by qPCR analyses. The comparison with cases where the baby has received antibiotics directly, not via IAP, indicated that IAP had an equal or even higher negative effect on the microbiota establishment in the infant. Later studies conducted with the same infant cohort showed that babies from mothers who had received IAP harbor lower abundances of Actinobacteria, higher levels of Firmicutes and Proteobacteria, and lower levels of acetate and total short-chain fatty acids in their gut microbiota^[16]^. Moreover, the study concluded that IAP may involve lasting effects on physiology, which could affect the neonatal microbiota-host crosstalk with long-lasting effects on health, due to the observed differences in inferred metabolic pathways affected by the IAP treatment. Similarly, in a study comparing the gut microbiota composition in meconium of a cohort of prematurely born babies with and without IAP and the vaginal microbiota of mothers, Zhou *et al.* observed that the *Lactobacillus* population was decreased after IAP in the mothers’ vagina and newborns’ meconium samples^[17]^. In addition, they observed specific alterations in meconium (*Staphyloccous* or *Sphingomonas *increment) from some IAP premature babies diagnosed with early-onset sepsis (EOS). This may indicate an association between EOS and antibiotic-mediated dysbiosis in the premature gut.

It is important to take into consideration that prematurely born babies are a cohort of neonates very exposed to medications and clinical practices due to their underdevelopment at birth. Thus, the gut microbiota establishment in those babies is a challenging process due to different factors surrounding organ immaturity, long stays at hospitals, medications, oxygen support, antibiotics, *etc.*, and, therefore, they may not be very representative of the situation in healthy full-term infants. For this reason, most of the studies focused on the effect of IAP on gut microbiota development have been performed in full-term infants since these represent a more common situation. Moreover, in most cases, the studies used vaginally delivered babies, although some studies have also included infants born by C-section [Table 1], in most cases observing different findings depending on delivery mode. One of the first studies published with full-term neonates was carried out in a cohort of Canadian babies, where the effect on the intestinal microbiota composition of penicillin (in vaginal deliveries) and cefazolin (in emergency and elective C-sections) were studied at three months of age^[18]^. Changes in the gut microbiota were observed, with depletion of Bacteroidetes and loss of diversity among the most significant results. Moreover, the relative abundances of *Bacteroides* and *Parabacteroides* genera were decreased, while *Enterococcus*, *Clostridium*, and Proteobacteria were increased after IAP in the three groups of babies analyzed^[18]^.

**Table 1:** Main demographic and technical characteristics of studies investigating the effect of IAP on gut microbiota development included in this review

**Study population** **(delivery mode/*n* [*n* in IPA group]/stool collection age)**	**Antibiotic**	**Technique for gut microbiota analyses**	**Refs.**
Full-term, vaginal and C-section/*n* = 50 [*n* = 23 and *n *= 2]/3 days	Amoxicillin	Culturing stool samples	[13]
Full-term, vaginal/*n* = 52 [*n* = 26]/7 days	Ampicillin	qPCR, PCR-DGEE	[14]
Very low birth-weight preterm (24-32 GW)/*n* = 27 [*n* = 14]/2, 10, 30, 90 days	Penicillin (*n* = 1), ampicillin (*n* = 1), or ampicillin + erythromycin (*n* = 12)	16S rRNA amplicon sequencing (V3, Ion Torrent), qPCR	[15, 16]
Full-term and preterm (< 37 GW) (vaginal and C-section)/*n* = 50 and 48 [*n* = 27 and *n* = 25]/meconium	Cefazolin	16S rRNA amplicon sequencing (V4, Illumina)	[17]
Full-term vaginal and C-Section/*n* = 189 [*n* = 42 and *n* = 43]/3, 12 months	Penicillin (vaginal), cefazolin (C-section)	16S rRNA amplicon sequencing (V4, Illumina)	[18]
Full-term, vaginal/*n* = 40 [*n* = 18]/2, 10, 30, 90 days	Penicillin	16S rRNA amplicon sequencing (V3, Illumina)	[19]
Full-term, vaginal/*n* = 149 [*n* = 44]/0, 1, 2, 3, 4 days, 6 months	Penicillin (*n* = 38), cefuroxime (*n* = 4), clindamycin (*n* = 2)	16S rRNA amplicon sequencing (V4-5, Ion Torrent), shotgun metagenomics (Illumina)	[20]
Full-term, vaginal and C-section/*n* = 74 [*n* = 14 and *n* = 7]/3, 10 days, 6, 12 weeks	Penicillin (vaginal); cefazolin (*n* = 5), ampicillin (*n* = 1) and penicillin (*n* = 1) (C-section)	16S rRNA amplicon sequencing (V3, Illumina)	[21]
Full-term, vaginal and C-section/*n* = 130 [*n* = 14]/1, 3, 6 months	Ampicillin (vaginal), cefazolin (C-section)	16S rRNA amplicon sequencing (V3-V4, Illumina)	[22]
Full-term, vaginal/*n* = 100 [*n* = 27]/12 months	Penicillin (*n* = 25), cefuroxime (*n* = 2),	16S rRNA amplicon sequencing (Ion Torrent PGM)	[23]
Full-term, vaginal/*n* = 266 [*n* = 87]/6 weeks, 12 months	Penicillin (*n* = 55), cephalosporin (*n* = 14), mix (*n* = 18)	16S rRNA amplicon sequencing (V4-V5, Illumina)	[24]
Full-term, vaginal/*n* = 26 [*n *= 13]/7, 30 days	Ampicillin	16S rRNA amplicon sequencing (V3-V4, Illumina), qPCR	[25]
Full-term vaginal and C-Section/*n *= 1654 [*n* = 375 and *n* = 403]/3 months	Penicillin (vaginal), cefazolin (C-section)	16S rRNA amplicon sequencing (V4, Illumina), qPCR	[26]
Elective C-Section/*n* = 44 [*n* = 44]/10 days, 9 months	Cefuroxime	16S rRNA amplicon sequencing (V3-V4, Illumina)	[27]
Full-term C-Section and vaginal/*n* = 63 [*n* = 40 and *n* = 23]/1, 7, 28 days, 3 years	Cefuroxime (C-section)	16S rRNA amplicon sequencing (V3-V4 first month, V4 3 years - Illumina), shotgun metagenomics (7, 28 days - Illumina)	[28]
Full-term, vaginal/*n* = 84 [*n* = 35]/7, 30 days	Ampicillin	qPCR	[29]
Full-term, vaginal and C-section/*n* = 43 [*n* = 7 and *n* = 9]/2, 10, 30, 90 days	Ampicillin (vaginal), cefazolin (C-section)	ITS (16S-23S) amplicon sequencing (Illumina), qPCR	[30]

IAP: Intrapartum antimicrobial prophylaxis; GBS: group B streptococcus; qPCR: quantitative PCR; PCR-DGEE: PCR-denaturing gradient gel electrophoresis; GW: gestation weeks; Ab: antibiotics.

The vast majority of studies focusing on full-term vaginally delivered babies showed important changes in the gut microbiota composition during the first months of life, with a lower number of studies with follow-up at later ages. Some alterations in the intestinal microbiota were also detected during the first days of life by different authors. In meconium samples of both premature and full-term babies from IAP mothers, lower levels of lactobacilli were observed^[17]^. At seven days of life, after penicillin prophylaxis during delivery, lower diversity and lower abundance of *Bifidobacteriaceae* (and significantly lower in genus* Bifidobacterium*), *Bacteroidaceae*, *Lachnospiraceae*, or *Lactobacillaceae* were observed, followed by enrichment in *Enterobacteriaceae*, *Clostridiaceae*, or *Streptococcaceae* in an Italian cohort of babies subjected to 16S rRNA gene sequencing^[14]^. Some of these observations were corroborated by Nogacka *et al.* in a Spanish cohort of vaginally delivered full-term babies at 10 days of age^[19]^. They showed higher proportions of Proteobacteria, *Clostridiaceae*, and S24-7 families in an IAP group of neonates and lower abundances of *Bifidobacteriaceae*. In agreement with these data, *Bacteroides* was also one bacterial group decreased while Proteobacteria and *Clostridium* increased during the first days of life in a group of four-day-old IAP babies from Finland^[20]^.

At one and three months of age, Nogacka *et al.* still continued detecting differences due to IAP treatment, with significant enrichment of *Campylobacteriaceae*,* Helicobacteraceae*,* Prevotellaceae*, and S24-7 families and lower levels of alpha-diversity, in general and specifically of *Bifidobacteriaceae*, in IAP babies^[19]^. Similar results were found by Stearns *et al.* in a Canadian cohort of vaginal full-term infants at six and twelve weeks, where lower microbial diversity and bifidobacterial abundances were observed, in addition to an increment of *Clostridium* and *Escherichia* genera^[21]^. A low abundance of *Bifidobacterium* was also detected in Japanese infants whose mothers were administered intrapartum antibiotics in both vaginal (ampicillin) and C-section (cefazolin) delivery^[22]^.

An examination of the impact of IAP on the gut microbiota beyond the third month of life has been conducted in a few cohorts of babies. While Imoto *et al.*^[22]^ did not observe any statistical differences in a group of six-month-old infants, Tapianien *et al.*^[20]^ pointed out that the effect of IAP could be comparable with that caused by direct postnatal antibiotics, with lower levels of *Bacteroides* still being present at six months of age. This cohort of babies was also examined at one year of age, and the authors found consistent results, with lower relative abundances of Bacteroidetes and *Bacteroides* and an increase of *Escherichia coli* in the IAP group^[23]^. Similarly, Azad *et al.* also observed alteration in the gut microbiota at one year of age in their Canadian cohort^[18]^. These were mainly characterized by higher relative abundances of *Clostridiaceae* in the vaginally delivered IAP group as well as in an IAP emergency C-section group where, in addition, lower diversity and proportions of *Bacteroides* were observed.

In 2020, a prospective study was carried out to evaluate the impact of the different specific classes of antibiotics administered as IAP. Lower abundance of *Bacteroides*, *Bifidobacterium Ruminococcus*, *Blautia*, and *Roseburia* and higher proportions of *Oscillospora* and *Veillonella* were found at both six weeks and one year of age after the exposure to any class of antibiotics^[24]^. In addition, the authors concluded that IAP alters the natural development and trajectory of the infant gut microbiome; its effects persist after one year of life, and particular alterations were associated with specific antibiotics. Over time, they observed a smaller increase in *Bacteroides* and *Bifidobacterium* and a rise in *Coprococcus* in infants exposed to penicillin. Cephalosporin entailed a smaller increment in *Bifidobacterium* and *Enterococcus*, and when a mix of antibiotics was used, a decrease in *E. coli* abundance was observed, in comparison with a non-IAP group of neonates. Moreover, differentially abundant functional metagenomes were also observed at one year of age^[24]^.

Breastfeeding is one of the most influential factors impacting gut microbiome development, and it is usually a confounder when the impact of other perinatal factors is questioned. Some of the studies focusing on IAP impact also examined the impact of breastfeeding on gut microbiota acquisition after perinatal antibiotics. Mazzola *et al.* studied the impact of IAP in breastfed and formula-fed infants and observed a different evolution of the gut microbiota during the first month of life^[25]^. IAP breastfed infants showed lower diversity and absence of *Bifidobacterium* at seven days and recovery at one month, but with a dominance of enterobacteria in their gut microbiota. However, IAP formula-fed infants showed a dominance of Bacteroidetes at one month, with respect to no IAP formula-fed and IAP breastfed infants. Nogacka *et al.* also suggested a differential response to IAP treatment depending on the feeding mode^[19]^. Azad *et al.* suggested a protective role of breastfeeding in babies exposed to IAP due to emergency C-sections and breastfed at least for three months^[18]^. However, in an extended Canadian cohort of babies^[26]^, the authors observed significantly lower proportions of *Bifidobacterium* in exclusively or partially breastfed, vaginally delivered IAP-treated infants. Those inconsistent findings reported by different studies indicate the need for more studies on the role of breastfeeding as a modulator of the IAP-induced alterations in the infant microbiota development process.

As stated above, few studies have focused specifically on the impact of IAP in C-section delivered babies. Apart from the information unveiled by Azad *et al.*^[18] ^with respect to the changes observed after IAP in emergency and elective C-sections, in the same year, Stearns *et al.*^[21]^ also included a group of C-section babies in their study. The authors concluded that IAP has an impact independent of delivery type in the gut microbiota development during the first three months of life, with a special negative impact on Bacteroidetes. Chen *et al.* also observed a severe depletion of Bacteroidetes and an increment in Firmicutes and Proteobacteria in C-section delivery after maternal IAP exposure^[26]^. Other studies aimed at unraveling the effect of IAP timing on C-sections have concluded that C-section delivery affects the gut microbiome colonization more strongly than antenatal antibiotic exposure^[27,28]^. These studies did not observe differences between the group of babies whose mothers received antibiotics prior to skin incision or those after umbilical cord clamping, with *Bacteroides* and *Bifidobacterium* most negatively affected and Proteobacteria increased at one month of life, but not at three years of age, with respect to a control group of vaginal-delivery babies^[28]^.

**Figure 1:**
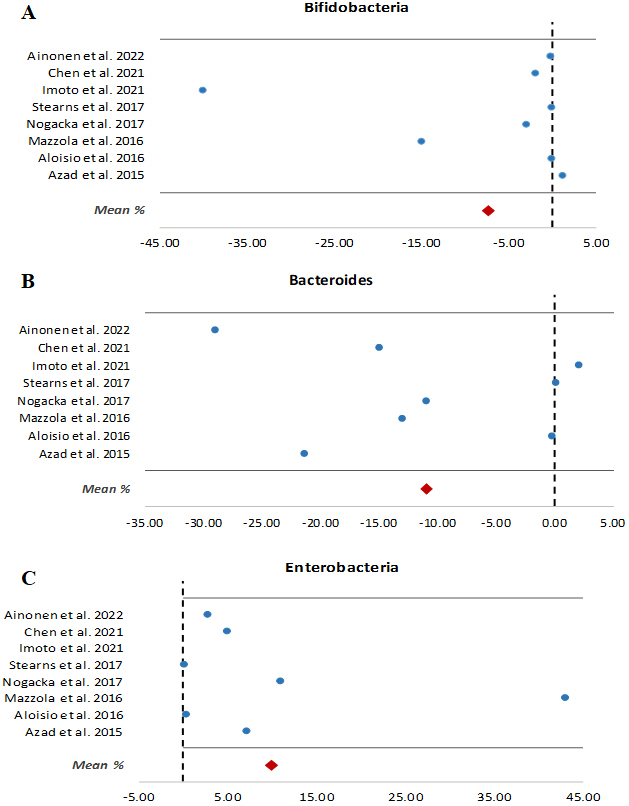
Changes in the relative abundances (%) as a result of IAP exposure in different studies of: (A) bifidobacteria; (B) bacteroides; and (C) enterobacteria. Data are from between 7 and 90 days of life, with the exception of the Ainonen *et al.*’s study^[23]^, which was conducted at one year in full-term vaginally delivery babies. Mean (%) was calculated from the relative abundances numerically available in the eight studies included. IAP: Intrapartum antimicrobial prophylaxis.

Most of the studies revealed that *Bifidobacterium* and *Bacteroides* are two of the taxa more vulnerable to maternal prophylactic antibiotics, although Imoto *et al.* found that a reduction in *Bacteroides* was more associated with C-section delivery than with IAP treatment^[22]^. It was observed that IAP reduced not only the relative abundances of genus *Bifidobacterium* but also its absolute quantities, as was confirmed by qPCR^[14,15,29]^. Moreover, not only at the genus level but also at the species level, the negative impact of IAP has been demonstrated on bifidobacteria populations by DGGE-PCR^[14]^ and ITS-sequencing^[30]^. A 7.2% relative abundance decrement of this genus per each hour of IAP against GBS in vaginal births was also observed by Stearns *et al.*^[21]^. Conversely, most studies showed that, in this dysbiosis situation, Proteobacteria increase in abundance. Figure 1, encompassing eight studies with relative abundances available in their respective manuscripts for bifidobacteria, bacteroides, and enterobacteria at genus or family level, shows how the two former taxa are negatively affected by IAP, while enterobacteria are increased in the gut microbiota of full-term vaginally delivered babies at 7-90 days old.

Long-term effects of IAP on the gut microbiota, further than those reported at one year of age, have not been published yet. However, a higher risk of cerebral palsy or bowel dysfunctions in children who were born prematurely and whose mothers were administered erythromycin were observed at the age of seven years^[31]^, while a relationship between IAP for GBS prophylaxis and BMI-Z scores was not detected in children at 2-5 years^[32]^. Beyond gut microbiota, some studies have also been published showing differences in the infant oral microbiota due to maternal intrapartum antibiotics^[33,34]^.

## IMPACT OF IAP ON THE DEVELOPING INFANT RESISTOME

As discussed above, different authors showed how IAP affects the initial establishment of the infant gut microbiota and how different bacterial groups vary as a function of this perinatal factor. However, little is known about the connection between IAP and the infant gut resistome since few authors have thus far looked into this matter. 

Nogacka *et al.* assessed the prevalence of 11 ARGs in one-month-old infants born at term by vaginal delivery^[19]^. The prevalence of the ARGs *bla*TEM, *CTX*-M, and *aac*6-*aph*2 was higher in the group of children whose mothers received prophylaxis. Among these three genes, *bla*TEM was present in 20% more infants in the IAP group than in the control group. A recent study suggested a potential detrimental impact of IAP on increasing the risk for harboring ARGs at one year of age, although delivery mode (C-section) was found to be an important confounding factor in infant resistome studies^[35]^. Tapiainen *et al.* studied not only the effect of IAP but also the direct administration of antibiotics to the newborn after birth and the resulting combination of both factors on infant gut microbiota^[20]^. Although their data in the resistome should be taken with caution, as the number of samples was low and the infants received a probiotic, the results show that this group of infants harbor an increased abundance of ARGs in their gut microbiota. The most commonly detected ARGs were related to the presence of species belonging to the genera *Escherichia *and *Staphylococcus. *They also analyzed the presence of ARGs in the gut microbiota of the mothers and found that they had a lower burden of ARGs compared to their offspring. The same cohort in Tapiainen’s study was also used by Li *et al.* to test whether these genes were vertically transmitted from mothers to infants^[36]^. Metagenomics analyses allowed them to identify the origin of the species carrying the resistance genes present in children’s feces by comparing mother and infant samples. The results show that vertical transmission decreased with the administration of antibiotics to both the mother and the newborn. The altered gut colonization caused by antibiotics led to the establishment of bacteria from the environment rather than from the mother’s microbiota. In this study, the resistance genes of most of the children in the antibiotic group came from species found in the hospital environment. In contrast, in the control group, the species carrying the genes were also found in the maternal gut microbiota.

In other works, ARG burden was directly related to the presence of certain bacteria, which could entail a direct risk to infants’ health, since the association of IAP with an increased presence of antibiotic-resistant bacteria in the intestinal microbiota of newborns, predisposing to late-onset bacterial infections, has been reported^[37,38]^. In fact, the use of IAP has been associated with an increase in GBS resistance to antibiotics such as clindamycin and erythromycin^[9]^. Along the same line, Pärnänen* et al.* determined that IAP increased the presence of ARGs and mobile genetic elements in the infant gut microbiota^[39]^. They associated a higher load of ARGs with higher counts of *E. coli* and different species of gamma-proteobacteria and with a decreased presence of bifidobacteria. Conversely, IAP has also been associated with increased ARGs not only in the gut but in other parts of the organism, such as the nasopharynx^[40]^. This study found that infants born to mothers treated with azithromycin had a higher prevalence of the *msr*A and *erm*C genes, which confer macrolide resistance, at 28 days of age. These genes were positively associated with the presence of AZI-resistant *Staphylococcus aureus. *Moreover, other authors have also reported a higher prevalence of *Vim*-1 (conferring β-lactamase resistance) in the oral microbiome of their cohort of babies exposed to IAP^[33]^.

Despite the different methodologies used, most authors agree that IAP alters the initial establishment of the intestinal microbiota, which may predispose infants to various pathologies and infections. This alteration, together with the selective pressure exerted by antibiotics, favors the presence of bacteria carrying antibiotic resistance genes.

## DISCUSSION

The data accumulated during the last decade clearly demonstrate that the use of IAP alters the early-life microbial colonization process, affecting the intestinal microbiota composition during these very important early days of life. Although some differences in the effects reported by different authors do exist, there are also some common and reproducible observations. These include the increased levels of enterobacteria and reduction of strict anaerobe commensals such as bifidobactera and bacteroides during the first months of life. The long-term persistence of these differences is a current matter of debate since few studies have addressed this aspect and the techniques more frequently used, mainly 16S rRNA gene profiling and qPCR, allow for a general microbiota composition overview but do not provide data on potential strain replacements and other effects at the strain level. Similarly, our understanding of the long-term effects on the health of these early IAP-induced microbiota alterations is still very limited.

Moreover, recent evidence also suggests a potential detrimental effect of IAP on the infant intestinal resistome. Although only few studies on this subject have been published to date, those available suggest an impact. In a context with increasing problems due to antimicrobials-resistant microorganisms, and given the potential role of the gut microbiota as a reservoir for ARGs, understanding the effects of early-life interventions on the ARG pool and level is of great interest. Metagenomic studies, allowing for resistome analyses, are becoming available in this area, and they will provide new insight into this aspect.

To conclude, the evidence underlined in this manuscript, together with the demonstrated efficacy of IAP for avoiding GBS infections in the neonate, points to the need for rational use of IAP. This highlights the importance of intervention strategies limiting the impact of IAP on the infant gut microbiota composition and resistome, and the resulting long-term health implications.

## DECLARATIONS

### Copyright

© The Author(s) 2022.
